# Current Management of Conjunctival Melanoma Part 1: Clinical Features, Diagnosis and Histopathology

**DOI:** 10.4274/tjo.galenos.2020.38096

**Published:** 2020-10-30

**Authors:** İrem Koç, Hayyam Kıratlı

**Affiliations:** 1 Hacettepe University Faculty of Medicine, Department of Ophthalmology, Ocular Oncology Service, Ankara, Turkey

**Keywords:** Conjunctival melanoma, diagnosis, prognosis

## Abstract

Conjunctival melanoma is a rare disease which makes up approximately 5% of ocular melanomas. The lesion may occur de novo or originate from a pre-existing nevus or primary acquired melanosis. Biomicroscopy is of paramount importance in diagnosis and follow-up of the disease, while other diagnostic modalities serve as supplementary tools. Many clinical and histopathological risk factors have been reported for prognosis. This review aims to address the clinical findings, differential diagnosis, diagnostic tools, prognostic factors, and staging of this disease.

## Introduction

Primary melanoma of the eye can occur in four different anatomical compartments of the globe: the orbit, eyelids, conjunctiva, and uvea (subdivided as the iris, ciliary body, and choroid). Conjunctival melanoma (CM) is considered an ocular surface neoplasia and accounts for 1-7% of all ocular melanomas, with an incidence rate nearly one-tenth of that of uveal melanoma in whites.^1^ CM, which can arise from primary acquired melanosis (PAM), from an existing conjunctival nevus, or de novo, is derived from a malignant proliferation of melanocytes of neural crest origin that normally reside in the basal layer of the conjunctival epithelium.^2^

CM differs substantially in histopathology, genetic profile, and management from other ocular melanomas and is handled as a separate entity in clinical practice. Even so, in terms of histopathogenesis, molecular biology, and biological behavior such as distant metastatic pattern, CM lies biologically closer to mucosal and cutaneous melanomas than does a uveal melanoma.^3^ The pattern of metastasis usually presents with spread to the regional lymph nodes first in CM and cutaneous melanoma, while uveal melanoma primarily tends to cause hematogenous metastasis to the liver.^4^ Another common trait between CM and cutaneous melanoma is that they are derived from melanocytes of neural crest origin, which migrate toward epithelium, whereas the melanocytes that form uveal melanoma cells migrate into deep mesodermal tissue.

CM is a potentially sight- and life-threatening tumor if left untreated, with a 10-year mortality rate up to 30%.^4^ Spread of the uncontrolled disease can manifest as local recurrence, involvement of distant conjunctiva, or distant metastasis through regional lymph nodes via involvement of blood vessels or lymphatics located in the substantia propria of the conjunctiva.^1^ All considered, CM requires appropriate management in line with the recent advances in our understanding of this disease.

## Epidemiology

The current epidemiological data for CM shows an incidence of 0.2-0.8 per million with a race predilection favoring non-Hispanic whites, and even though it is a rare disease, there is an upwards trend in incidence which is mostly attributed to ultraviolet radiation exposure.^5^ It is a disease of middle-aged individuals 55 to 65 years old, and is even rarer in childhood, where less than 1 in 20 conjunctival tumors are malignant. There is no proven sex predilection for CM. In large series of conjunctival specimens in tertiary referral centers, CM constitutes 12-25% of all excised conjunctival tumors and 23-25% of all excised melanocytic conjunctival lesions.^6^ Population-based studies report a lower ratio, including a recent study from Olmsted County, Minnesota reporting 6 melanoma cases out of 504 patients with a conjunctival tumor.^7^ Table 1 shows the incidences reported for CM in recent population-based studies involving Caucasian populations in the Western world.^3,7,8,9,10,11,12^

## Clinical Findings

**Figure 1:**
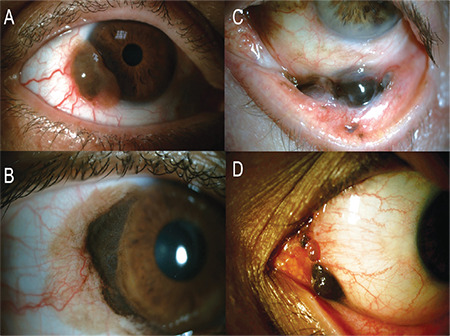
Anterior segment photography of various clinical presentations. A) A limbal, vascularized melanotic mass later proved to be CM. Note the finely vascularized amelanotic base and dilated conjunctival feeder vessels. B) A large limbal CM surrounded by diffuse PAM, which suggests PAM as the origin. C) Forniceal location of CM. D) CM involving the plica semilunaris and caruncle.

CM clinically presents as an elevated macule, plaque, nodule, or diffuse infiltration with varying pigmentation from light brown to dark brown, and in rare cases as an amelanotic mass (Figure 1). Recurrent lesions tend to be lighter compared to primary CM.^13^ Any immobile, melanocytic conjunctival lesion with prominent vascularity should raise suspicion for CM. Nearly one third of CMs can be multifocal.^14^ The most common location for CM is the peribulbar conjunctiva near the limbus, especially temporally.^4^ Benign tumors are rarely seen in the extrabulbar conjunctiva (palpebral and forniceal conjunctiva) and caruncle, so any pigmented lesion in this area should raise suspicion for CM.

## Differential Diagnosis

The differential diagnosis of CM includes other melanocytic lesions of the conjunctiva including conjunctival nevus, congenital melanosis, primary or secondary acquired melanosis, extraocular extension of uveal melanoma, metastatic CM of cutaneous origin,^15^ pigmented squamous cell carcinoma or papilloma, sebaceous carcinoma, and oncocytoma. A variety of non-melanocytic entities such as Axenfeld nerve loop, pyogenic granuloma, infected epithelial inclusion cyst, post-surgical hematoma, mycosis, mascaroma, argyrosis, pinguecula, and foreign body are the other entities that can mimic CM. In all patients (including pediatric cases), older age, larger mean basal diameter, thicker tumors, hemorrhage, and absence of cysts favor CM rather than conjunctival nevus.^6^ The co-occurrence of an intraocular tumor and a pigmented conjunctival lesion with spared conjunctival epithelium should initially suggest an extraocular extension of the intraocular tumor, because CM only invades the globe in the most advanced cases unless there is a facilitating wound such as previous sclerectomy or cataract incision.^16^ Of the entities considered for differential diagnosis, PAM is of particular clinical importance and will be addressed separately below.

## Primary Acquired Melanosis

**Figure 2:**
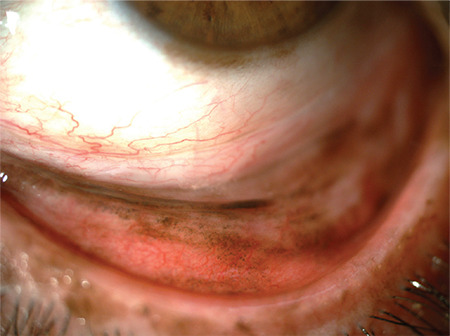
Eversion of the lower eyelid reveals diffuse PAM, especially in the tarsal conjunctiva. Note the additional limbal pigmentation.

PAM is considered the benign counterpart of CM and is the precursor lesion in 25-75% of CMs, while nearly 50% of PAM with atypia progress to CM.^17^ PAM usually presents as unilateral patchy or diffuse superficial pigmentation of the epibulbar conjunctiva with or without waxing and waning (Figure 2). The rest of CM cases not associated with PAM arise from preexisting nevi or de novo, with only 1% of conjunctival nevi found to progress to CM after 7 years of follow-up.^2,15^ Additionally, dysplastic nevus syndrome is another possible predisposing condition for CM, although a risk prediction or prognostication for CM or a direct link between the two diseases has yet to be determined. Studies of time trends in CM incidence have revealed that CM lesions have been detected at lower thicknesses and diameters over time, which suggests earlier diagnosis, but tumors as large as 40 mm in largest basal diameter and 15 mm in thickness are still reported in large series.^6^

In general, CM presents nearly a decade later than PAM, which correlates with the process of transformation into melanoma. Similarly, patients with PAM without atypia were found to be younger than those having PAM with atypia, though a similar pattern of progression among the latter two has yet to be determined in humans.^17^ The differentiation of PAM with atypia and without atypia is based on histopathology only; however, clinical clues favoring CM versus PAM have been defined as: thickness more than 1 mm, lack of pigment, presence of feeder or intrinsic vessels, cysts, hemorrhage, older age, and tarsal location.^6^

The terminology for what is called PAM today has shifted over time, initially from precancerous melanosis to benign acquired melanosis, then to melanoma in situ,^18^ and today PAM represents acquired melanosis with or without atypia.^16,17^ Some centers have replaced the term PAM with conjunctival melanocytic intraepithelial neoplasm (C-MIN) with an additional histological grading system based on horizontal epithelial involvement, vertical depth of melanocytic infiltration, and degree of cellular atypia, where the lowest C-MIN score of 0 corresponds to melanosis, 1 corresponds to “PAM with mild atypia”, 2 or 3 corresponds to “PAM with moderate atypia”, 4 corresponds to “PAM with severe atypia”, and a score of 5 or more corresponds to CM in situ.^19^ In both cases, either PAM or C-MIN, the lesions are described clinically as flat, usually unilateral, patchy or diffuse, unifocal or multifocal, noncystic melanocytic lesions of the conjunctiva generally seen in Caucasians.

Based on current knowledge, it is now considered overtreatment to perform orbital exenteration, whereas this was once considered the main approach for these lesions.^18^ The contemporary approach to PAM treatment lacks standardization and consists of close observation, excision alone or combined with cryotherapy, and topical chemotherapy with mitomycin C, 5-fluorouracil, or interferon-alpha-2-beta.^19^ Additional mapping biopsies before commencing treatment may aid in determining the need for brachytherapy in tumors with a high C-MIN score, the extent of the disease (particularly in amelanotic disease),^19^ and the degree of atypia at different locations, as the lesion may be multifocal. However, incisional or needle biopsies for CM should be avoided because these procedures are associated with tumor recurrence and iatrogenic seeding.^1^ Impression cytology (IC) is also not recommended in melanocytic proliferations of the conjunctiva.^20^ Our approach to PAM/C-MIN consists of total excision where possible and several incisional biopsies combined with topical 0.04% mitomycin C drops 4 times a day for 2 weeks followed by a 2-week drop-free period, for at least 2 cycles, targeting residual or resistant areas in more extensive cases. Extensive PAM or PAM with atypia should be approached with vigilance, as PAM with atypia has a 13% risk of conversion to CM as opposed to 0% in PAM without atypia, and each clock hour increase in the extent of the lesion increases the likelihood of CM 1.7 times.^21^ Histologically, PAM with atypia is considered to pose a higher risk of progression to melanoma with increasing number of epithelioid melanocytes and when there is intraepithelial pagetoid spread.^16^ Additionally, the risk of progression to CM increases with increasing clinical extent of PAM.^21^

## Histopathology

Detecting the atypical melanocytes of CM can be challenging, particularly when composed purely of small polyhedral cells. The pathological diagnosis of CM is made when atypical melanocytes are seen to invade the substantia propria, with loss of maturation and loss of normal polarity.^15^ CM can be composed of 4 different cell types in variable proportions: small polyhedral cells, epithelioid cells, balloon cells, and spindle cells.^17^ Features that favor melanoma rather than nevus histopathologically are intraepithelial component of PAM with atypia displaying pagetoid growth, intraepithelial radial extension beyond the lateral edge of invasion of the substantia propria, inflammation at the base of the lesion, mitotic activity, loss of normal polarity, and production of tyrosinase at the base of the lesion.^16^ Invasion of the substantia propria is required for definitive diagnosis of CM; however, in both PAM and CM, atypical melanocytes can show nesting in the epithelial junction and pagetoid spread into the epithelium, where prominent atypical features such as nuclear pleomorphism, prominent nucleoli, atypical mitoses, and abundant cytoplasm favor CM.^16^

## Diagnostic Tools

As for all conjunctival lesions raising suspicion for malignancy, the gold standard for CM diagnosis relies on histopathology.^22^ However, there are some adjuvant diagnostic tools which aid in differential diagnosis.

### a) Slit-lamp Biomicroscopy Documentation and Follow-up

Photographic documentation of the conjunctival melanocytic lesion should be carried out initially and at follow-up visits together with clinical mapping, expression of extent in clock minutes, and schematic drawing introduced by Damato et al.^23^ A thorough slit-lamp examination and documentation of the following should be made at every visit: the eyelid skin, whole conjunctival surfaces including palpebral conjunctiva with eversion, forniceal conjunctiva, tarsal conjunctiva, caruncle, plica, puncta, and all other visible portions of bulbar and nonbulbar conjunctiva. Care should be taken to note corneal involvement, if any. Adherence of the tumor to the underlying structures should be tested using a cotton-tipped swab as it is both helpful in differential diagnosis and surgical planning. The preferred approach is to excise the lesion in total when CM is suspected; however, serial photographic and schematic documentation is crucial when monitoring a PAM lesion since it is not possible to clinically determine which PAM lesions will transform into CM.

### b) Impression Cytology

There are few studies on IC of pigmented conjunctival lesions, including CM.^24,25^ Of these, Paridaens et al.^25^ reported 74% agreement between IC and histopathology in a 24-patient series including 9 cases of CM. Eight cases of CM were suitable for IC evaluation, and in 7 out of 8 cases IC confirmed later biopsy-proven CM, and 4 out of 7 cases of PAM with atypia were diagnosed accurately with IC.^25^ Keijser et al.^24^ reported 85% sensitivity, 78% specificity, 59% positive predictive value, and 93% negative predictive value for IC for conjunctival pigmented lesions in a study of 294 smears and 157 histological samples from 182 patients. They suggested prompt excision for grade 3 and 4 lesions in IC, but CM also developed within 6 months after IC in 35% of grade 0 lesions (insufficient material for diagnosis), 6% of grade 1 lesions (normal conjunctival cells), and 7% of grade 2 lesions (melanocytes with mild atypia).^24^ In the light of these findings, because IC only evaluates superficial epithelial cells, leaving out deeper lesion components, and morphological changes could be induced in the samples with brush cytology, IC is not recommended in the current or 8^th^ edition of the American Joint Committee on Cancer (AJCC) guideline.^20^

### c) Dermoscopy

Dermoscopy is a method of in vivo microscopic visualization of particularly pigmented skin lesions. Recently in a series of dermoscopic visualizations of 147 conjunctival lesions, 8 were CM with brown pigmentation, and the defined dermoscopic pattern for these was irregularly distributed dots confluent in a structureless pattern.^26^ In differentiation of CM from PAM, PAM lesions in this series had a diffuse distribution of dots, and as an indication of uninvolved episclera and sclera in the pigmented conjunctival lesions, they observed and described a “flag sign” of multiple epithelial folds of the pigmented lesion at the edge of the lesion.^26^ Whether dermoscopy and digital surface dermoscopy would be complementary imaging for slit-lamp biomicroscopy remains controversial due to small sample sizes.

### d) Pump-probe Microscopy

Pump-probe microscopy uses a two-colored pulse laser source to distinguish between different types of melanins with high spatial resolution. Wilson et al.^27^ demonstrated qualitative and quantitative differentiation of melanin composition in conjunctival nevi, PAM, and CM.^27^ The authors believed this imaging method aided in the detection of recurrences and evaluation of surgical margins by taking advantage of biological and photochemical properties.^27^ Similarly, Robles et al.^28^ reported 92.3% and 97.5% sensitivity and specificity, respectively, in the differentiation of invasive pigmented conjunctival lesions from noninvasive counterparts with this method.^28^

### e) Anterior Segment Optical Coherence Tomography (AS-OCT)

Anterior segment optical coherence tomography is superior to ultrasound biomicroscopy (UBM) imaging in terms of visualization of anterior chamber anatomy and imaging of the anterior border of the lesion, but it fails to demonstrate posterior layers of larger and pigmented tumors due to optical shadowing. With high-resolution OCT, which has increased scan depth and axial resolution, conjunctival nevi and CM are shown to display intensely hyperreflective basal epithelial layers, and CM is differentiated from nevi by the intense posterior shadowing seen with most CMs.^29^

### f) *In vivo* Confocal Microscopy (IVCM)

*In vivo* confocal microscopy is an anterior segment imaging modality which utilizes near-infrared laser to collect the reflection at the same point as the light source. Its use is limited in CM since it provides no sense of depth or thickness, which is crucial in management of the disease. In CM, IVCM can display atypical, highly reflective cells with prominent nuclei and large nucleoli, such as in extrascleral extension of uveal melanoma. It can also be helpful to differentiate between PAM with and without atypia, as PAM with atypia shows a large network of dendritic cells and hyperreflective granules throughout the epithelium, while these are confined to the basal layer in PAM without atypia. As a diagnostic tool for CM, IVCM is reported to have 100% sensitivity and 78% specificity for diagnosis of conjunctival malignant tumors.^30^ With IVCM, the presence of hyperreflective Langerhans cells mimicking malignant melanocytes is considered the main cause for misdiagnosis of malignant conjunctival tumors.^30^

### g) Ultrasound Biomicroscopy (UBM)

The use of UBM in CM as a diagnostic tool involves defining the extent and thickness of the disease, visualizing the tumor margins, and ruling out intraocular invasion of CM or extraocular extension of uveal melanoma. Even though UBM provides better resolution of pigmented conjunctival lesions with less optical shadowing and offers a larger field of view compared to AS-OCT, the depth of penetration is still limited to 4-5 mm due to high-frequency transducers.

### h) Photoacoustic Imaging

Photoacoustic imaging in vivo was recently described as a noninvasive tool for CM detection and growth monitoring in an animal model of CM in albino mice.^31^ The principle relies on the photoacoustic signal intensity of melanin and the purpose is to perform a photoacoustic tomography. The authors concluded that the photoacoustic signal correlated well with total and melanotic tumor volume.^31^ Still, this imaging method needs to be further confirmed in different clinical settings in human eyes with tumors of variable pigmentation and melanin content.

### i) Metastatic Screening and Systemic Work-up

Upon clinical examination of CM, suspicion of deep invasion of the sclera or intraocular, orbital, or sinus invasion should prompt computer tomography (CT) or magnetic resonance imaging (MRI).^20^ Anterior chamber angle invasion should readily be examined with gonioscopy and UBM is helpful in visualization of the anterior portion of the globe including the ciliary body and most anterior part of the sclera. Bowman’s membrane acts as a natural barrier against deeper invasion of the cornea, so care should be taken to leave the membrane intact during surgical excision. Invasion of the nasolacrimal passage and any other surrounding tissue by CM is also possible with pagetoid spread. Because the substantia propria of the conjunctiva is loose connective tissue rich in blood vessels and lymphatics, metastasis of CM can occur via lymphatic or hematogenous routes. Primary sites for lymphatic metastasis are regional draining lymph nodes of head and neck, including the preauricular, posterior auricular, submandibular, and cervical lymph nodes.^32^ Hematogenous dissemination can occur in virtually any part of the body but the most common sites for distant hematogenous metastasis are the lungs, brain, liver, and bones.^32,33^ Distant metastases without involvement of regional lymphatics are not uncommon.^33^ Accordingly, metastatic follow-up with annual chest X-ray and cranial MRI can be recommended in CM. The metastatic screening protocol of CM consists of clinical evaluation of the head and neck lymph nodes, liver function tests, liver ultrasound, chest X-ray, sentinel lymph node biopsy for lesions >2 mm with ulceration, and questioning nose bleeding, epiphora, change in smell sensation, and nasal obstruction, all repeated semi-anually.^34^ The role of positron emission tomography/computed tomography (PET/CT) has been limited in CM. Currently it is not suggested as a preoperative metastasis screening modality but rather a helpful tool in follow-up or restaging of selected patients.

### j) Sentinel Lymph Node Biopsy (SLNB)

In CM, the concept of regional metastasis in draining lymph nodes as a precursor of distant metastasis is partially invalidated by the fact that distant metastasis can occur without any clinical involvement of the lymph nodes.^3,33^ Still, the micrometastatic state of the regional lymph nodes remains to be tested in these cases. The estimated cumulative incidence of 10-year lymph node metastasis in CM is between 11% and 28%, and 45% of those who develop metastasis of CM have initial metastasis to lymph nodes.^32^ The rationale of SLNB is detection of lymphatic metastasis before it is clinically overt, assuming the patient will benefit from lymph node excision in terms of survival. In 2008, Tuomaala and Kivelä^35^ proposed a guideline to determine the CMs deserving SLNB, in which they suggested performing SLNB on tumors with >2 mm thickness and nonlimbal location. Their suggestion was based on the evidence that the cumulative incidence of initial or systemic metastasis of tumors measuring no more than 2 mm in thickness was 5% at 10 years and nearly 20% at 5 years for tumors with >2 mm thickness, and the cumulative incidence of initial or systemic metastasis of limbal tumors was less than 10% at 10 years and nearly 20% for nonlimbal tumors at 5 years.^35^ They performed SLNB at the time of excisional surgery.^35^ In 2015, Aziz et al.^36^ expanded the high-risk clinical and pathological characteristics that warrant SLNB to nonlimbal location, thickness >2 mm, ulceration on pathology, and >1 mitotic figures. Histopathologically, ulceration means the loss of epithelium over the tumor and is shown to be related to both lymph node and distant metastases. Therefore, SLNB should also be considered in tumors showing ulceration even when the thickness is <2 mm. Intraoperative SLN assessment is recommended by some groups based on the proposal of better visualization of lymph nodes intraoperatively, but objected to by others to avoid a possible iatrogenic tumor dissemination, or to allow time for detailed histopathological evaluation for high-risk factors.^36,37^ When a positive SLN is detected, neck dissection should be planned and depending on the extent of the disease, adjuvant therapy in the form of radiation treatment, chemotherapy, high-dose interferon, or biochemotherapy may be offered.^33^ The positivity rate with SLNB in CM is 11% to 16%, and the reported false-negativity rate (i.e., the development of nodal metastasis during follow-up despite exclusion of micrometastasis with SLNB) is as low as 8%. However, a consensus is lacking on the definition of false-negativity in terms of duration of follow-up.^37^ A recent study favoring SLNB was performed by Esmaeli et al.^38^ where 31 of 88 consecutive patients underwent SLNB and positive SLN was significantly associated with worse disease-free survival. The authors concluded that SLN positivity was a strong predictor of prognosis and therefore SLNB is helpful in the classification of high-risk patients and nomination of those who will receive adjuvant treatment.^38^

## Prognosis

To date, a large number of population-based or clinical studies have reported local recurrence rates, 5-year and 10-year survival rates, risk factors for local recurrence, and risk factors for distant metastasis. These factors, described mostly in the last decade, can be classified as clinical and histopathological.

### a) Clinical

Local recurrence rates, mortality rates, and clinical factors associated with disease prognosis are listed in Table 2.^2,3,9,10,14,32,39,40,41,42,43,44,45,46^ A recent publication of 70 patients associated light iris color and low tumor pigmentation, and low tumor pigmentation was found to be related to metastasis formation and death in both uni- and multivariate analyses.^47^ Recurrence was associated with low tumor pigmentation in multivariate but not univariate analysis.^47^ This was confirmed by a larger series of 444 CM patients, including 177 recurrent cases, in which low primary tumor pigmentation was linked to higher recurrence rate, recurrences with low pigmentation, and greater risk of metastases and death; however, recurrences with low pigmentation did not carry risk for increased metastases or death.^13^

### b) Histopathological

Histopathological criteria for CM associated with worse prognosis and increased mortality are presence of tumor-associated lymphangiogenesis, lymphocytic infiltration of the tumor, tumor thickness more than 2 mm, presence of surface ulceration, increasing depth of invasion, absence of complete surgical clearance, mitotic figure count >5/10 high-power fields, pagetoid growth pattern, and absence of focal inflammation.^17^ Origin of the CM has no direct effect on prognosis but CM arising from PAM has a tendency to recur.^17^ The recurrence rate after excision of PAM with tumor-free surgical margins has been reported to be 26% in 5 years and 65% in 15 years.^2^

On a molecular level, chemokines and chemokine receptors have been studied as potential trophic factors for metastatic spread of several malignancies, including conjunctival melanoma.^48^ Immunoreactive scores for chemokine receptors CXCR4 and CCR10 were shown to be related to progression of melanocytic conjunctival lesions towards CM with significant differences in nevi versus melanoma, and CXCR4 upregulation was found to be related to metastatic potential of CM.^48^

## Staging

In order to clinically and histopathologically classify CM, a few decades ago the Clark-McGovern classification of cutaneous melanoma was adapted with partial success and certain limitations, mainly because the conjunctiva lacks a papillary dermis, unlike skin, and vertical growth of the lesion cannot be assessed properly. On a macroscopic level, the disease can be classified as focal/nodular or diffuse/widespread. Unlike CM without PAM, CM with PAM mostly exhibits other risk factors for metastasis such as melanocytic atypia and palpebral conjunctiva involvement; therefore, CM with and without PAM should be classified separately.

The most recent AJCC tumor (T), node (N), metastasis (M) classification system offers a classification for CM based on tumor location and size (T), lymph node status (N), and presence of metastasis (M) (Table 3).^20^ In this classification system, lower T grades correlate with less extensive disease, and according to the 7^th^ edition of the AJCC TNM classification, CM survival was found to correlate with local recurrence, lymph node metastasis, and death, with T1 tumors representing less risk than T2 and T3 disease.^49^ The term Tis, standing for melanoma in situ, was first introduced in the 7^th^ edition, together with separation of caruncular tumors from the rest of the nonbulbar locations, and further modifications made in the latest 8th edition include regarding Tis as a pathological diagnosis, reclassification of the depth of substantia propria invasion with a threshold of 2 mm in pathologic staging, and removal of biopsy criteria from the N0 category.^20^ It is also advised that the term PAM should also be used clinically and the underlying process, whether melanosis or melanocytosis, together with the extent should be reported pathologically.^20^

Validation of the 8^th^ edition of the AJCC classification of CM was conducted in a large-scale, multicenter international study including 288 eyes of 288 patients. The study confirmed higher mortality rates in cT2 and cT3 tumors than in cT1 as well as higher mortality rates in pT2 and pT3 tumors compared to pT1.^50^ Furthermore, tumor thickness, ulceration, and tumor invasion but not caruncle or plica involvement were identified as independent risk factors for mortality.^50^ Despite having a large cohort for such a rare cancer, this study lacked subgroup analysis and considered metastasis equivalent to mortality. Esmaeli^51^ further emphasized pathologic factors such as tumor thickness and ulceration as prognostic predictors and suggested incorporating these factors into the AJCC classification. A detailed subgroup analysis of cumulative mortality between pT1a, pT1b, pT2a, and pT2b was also recommended in order to study the exact effect of tumor thickness on mortality.^51^ Other authors have expanded the factors to be incorporated into future AJCC classifications by adding positive SLNB as a prognostic factor.^36,49^ Tumor thickness and histologic ulceration were reported as the strongest predictors for nodal metastasis, distant metastasis, and melanoma-related death, rather than bulbar versus nonbulbar location and caruncular versus noncaruncular location.^38^ This is in part due to the discrepancy between clinical and pathological classification of T-categories, where Tis can correspond to a broad range of clinical T-categories, T1, T2, and even T3.^38^ This problem is overcome by excluding Tis from metastasis analyses, which made it possible to purely study the effects of thickness and ulceration, as Tis lesions are not expected to cause distant metastases or death.^38^ Overall, it is likely that tumor thickness and ulceration will be more distinctively incorporated in future AJCC classifications, similar to that of cutaneous melanoma.

## Conclusion

Even though CM is regarded as a rare entity, it can have a severe impact on overall survival. Notably, the incidence rates are reported to show an increasing trend in some series. Biomicroscopy is indispensable in diagnosis, determination of additional features, and follow-up of the disease, whereas other imaging modalities can be used with their own limitations as adjunct tools. Metastatic work-up and SLNB should be conducted for the indications proposed in the literature. Staging is still in progress as new prognostic factors are defined to develop more precise indicators for overall survival.

## Figures and Tables

**Table 1:**
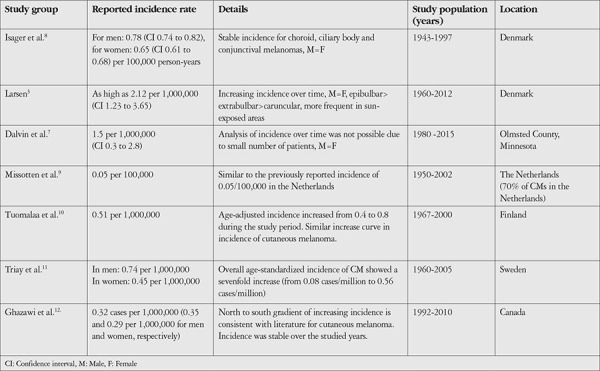
Recent population-based studies of CM involving Caucasian populations in the Western World

**Table 2:**
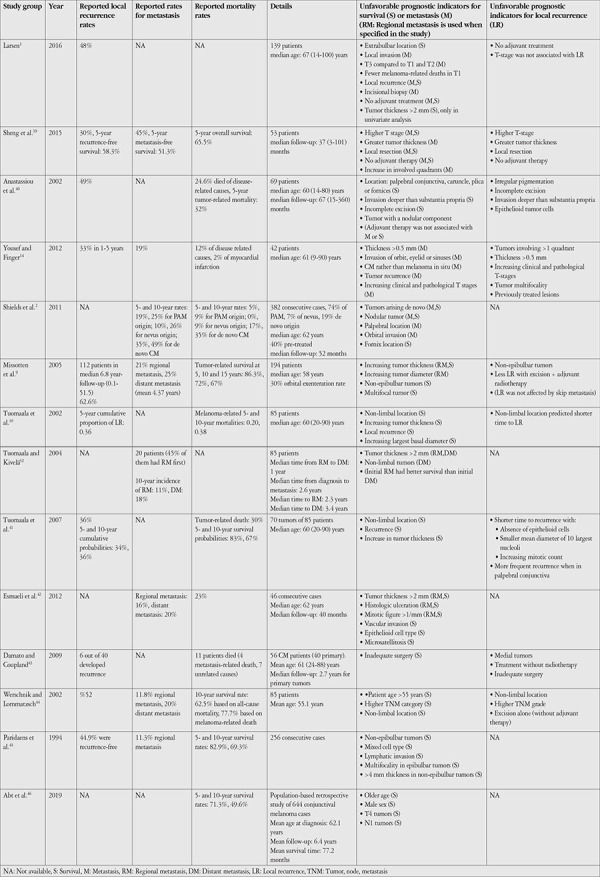
Local recurrence, metastasis, and survival rates, prognostic factors of CM reported in the literature

**Table 3:**
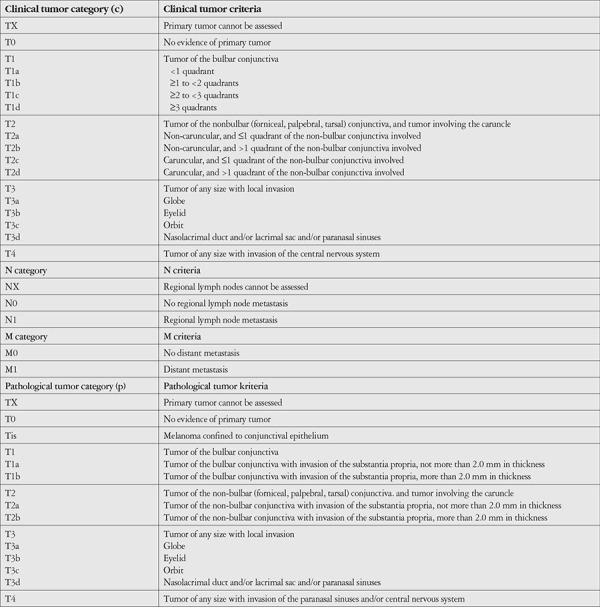
TNM definitions according to 8^th^ American Joint Committee on Cancer (AJCC) Classification for CM.^20^
